# An Intrinsic Mitochondrial Pathway Is Required for Phytic Acid-Chitosan-Iron Oxide Nanocomposite (Phy-CS-MNP) to Induce G_0_/G_1_ Cell Cycle Arrest and Apoptosis in the Human Colorectal Cancer (HT-29) Cell Line

**DOI:** 10.3390/pharmaceutics10040198

**Published:** 2018-10-23

**Authors:** Bee Ling Tan, Mohd Esa Norhaizan, Lee Chin Chan

**Affiliations:** 1 Department of Nutrition and Dietetics, Faculty of Medicine and Health Sciences, Universiti Putra Malaysia, Serdang 43400, Selangor, Malaysia; tbeeling87@gmail.com; 2 Laboratory of Molecular Biomedicine, Institute of Bioscience, Universiti Putra Malaysia, Serdang 43400, Selangor, Malaysia; 3 Research Centre of Excellent, Nutrition and Non-Communicable Diseases (NNCD), Faculty of Medicine and Health Sciences, Universiti Putra Malaysia, Serdang 43400, Selangor, Malaysia; 4 Department of Microbiology, Faculty of Biotechnology and Biomolecular Sciences, Universiti Putra Malaysia, Serdang 43400, Selangor, Malaysia; chanleechin@gmail.com

**Keywords:** c-Jun N-terminal kinase 1, cytochrome c, cytotoxicity, inducible nitric oxide synthase

## Abstract

Magnetic iron oxide nanoparticles are among the most useful metal nanoparticles in biomedical applications. A previous study had confirmed that phytic acid-chitosan-iron oxide nanocomposite (Phy-CS-MNP) exhibited antiproliferative activity towards human colorectal cancer (HT-29) cells. Hence, in this work, we explored the in vitro cytotoxicity activity and mechanistic action of Phy-CS-MNP nanocomposite in modulating gene and protein expression profiles in HT-29 cell lines. Cell cycle arrest and apoptosis were evaluated by NovoCyte Flow Cytometer. The mRNA changes (cyclin-dependent kinase 4 (*Cdk4*), vascular endothelial growth factor A (*VEGFA*), c-Jun N-terminal kinase 1 (*JNK1*), inducible nitric oxide synthase (*iNOS*), and matrix metallopeptidase 9 (*MMP9*)) and protein expression (nuclear factor-kappa B (*NF-κB*) and cytochrome c) were assessed by quantitative real-time polymerase chain reaction (PCR) and western blotting, respectively. The data from our study demonstrated that treatment with Phy-CS-MNP nanocomposite triggered apoptosis and G_0_/G_1_ cell cycle arrest. The transcriptional activity of *JNK1* and *iNOS* was upregulated after treatment with 90 μg/mL Phy-CS-MNP nanocomposite. Our results suggested that Phy-CS-MNP nanocomposite induced apoptosis and cell cycle arrest via an intrinsic mitochondrial pathway through modulation of Bax and Bcl-2 and the release of cytochrome c from the mitochondria into the cytosol.

## 1. Introduction

Colorectal cancer has become the third leading diagnosed malignancy and the fourth most prevalent cancer-associated death worldwide [1]. Total cases are projected to increase about 1.36 million and 1.08 million in 2035 worldwide for men and women, respectively [1]. Although tremendous efforts have been made to improve the available therapeutic approach, conventional therapy seems ineffective due to undesirable outcomes. Therefore, there is an unmet need to discover new anticancer agents with high efficacy but showing less adverse effects.

Phytic acid is a natural antioxidant found in vegetables, cereals, natural oils, and nuts. Phytic acid has numerous pharmacological properties such as anti-inflammatory, antioxidant, and anticancer activities [2,3]. Numerous animal and human studies have reported that phytic acid could prevent and suppress the tumorigenesis [4] via modulation of different molecular mechanisms such as apoptosis induction and reactive oxygen species (ROS) generation [4]. Although phytic acid has tremendous potential as a therapeutic agent for cancer, the effectiveness is limited by its shortcomings; for instance, low solubility, instability, and very short plasma half-life. Thus, one of the alternative ways to overcome this problem is through the application of nanotechnology [5].

Nanotechnology has emerged as a promising drug delivery system [6,7]. Application of nanotechnology could enhance the anti-cancer properties and improve bioavailability by increasing solubility, enhancing plasma half-life, and increasing permeation of the small intestine [8]. Due to their functional properties and feasible in characterization, magnetic nanoparticles show great potential in drug delivery systems [9]. Iron oxide nanoparticles (general formula: Fe_3_O_4_), are being applied in magnetic cell separation [10], drug delivery [11], cell labelling [12], and tumor labelling [13]. Emerging evidence has demonstrated the outstanding properties of iron oxide magnetic nanoparticles (MNPs) such as superparamagnetism and high specific surface area [14], and thus has been identified to be implemented in the medical field for diagnosis. However, iron oxide nanoparticles tend to form large aggregates owing to the strong magnetic dipole–dipole attractions between the particles. To overcome its instability and reduce the toxic effect, the iron oxide nanoparticle is usually encapsulated with polymers or surfactants [15]. Despite both natural and synthesized macromolecules have been applied to stabilize the iron oxide nanoparticles, polysaccharides and natural macromolecules offer great potential due to biodegradability and biocompatibility. Chitosan (CS) has been used as a mucoadhesive polymer due to its low toxicity, cost-effectiveness, and biodegradability [16]. CS shows the ability to enhance the bioavailability of orally administered drugs and increase the cellular permeability. A previous study demonstrated the efficacy of chitosan nanoparticle in drug uptake for therapeutic targets [17]. Nanoformulation using chitosan has been shown to increase a sustained release of the drug, adhere to gastrointestinal tract for a longer time, and increase retention time [18].

In our earlier study, phytic acid was successfully encapsulated with chitosan-iron oxide (hereinafter referred Phy-CS-MNP) which demonstrated good physiochemical properties including high thermal stability and encapsulation efficiency [11]. Indeed, an in vitro study has confirmed that Phy-CS-MNP nanocomposite exhibited antiproliferative activity towards human colorectal cancer (HT-29) cells. Hence, in this work, we explored the in vitro cytotoxic activity and mechanistic action of Phy-CS-MNP nanocomposite in modulating the expressions and key protein markers in HT-29 cells. This nanoformulation using iron-oxide-chitosan based nanocomposite will enhance the ability to release phytic acid for a longer time, which is important for phytic acid delivery purposes.

## 2. Materials and Methods 

### 2.1. Chemicals and Reagents

Phytic acid sodium salt from rice (≥90% purity) and chitosan (deacetylated at 75–85%) were bought from Sigma-Aldrich (St. Louis, MO, USA). Trypsin EDTA (1×), Dulbecco′s Modified Eagle Medium (DMEM), RPMI-1640 medium, Mycoplex™ fetal bovine serum (FBS), and penicillin and streptomycin (100×) were bought from Gibco (Grand Island, NY, USA). Bax and Bcl-2 Human SimpleStep Elisa^®^ Kits were procured from Abcam (Cambridge, UK).

### 2.2. Synthesis of Iron Oxide Nanoparticles 

The MNP was produced by co-precipitation method [19]. Briefly, MNPs were prepared by mixing 0.99 g of ferric chloride hexahydrate, 2.43 g of ferrous chloride tetrahydrate, 6 mL of ammonia, and 80 mL of deionized water. It was stirred for 1 h prior to centrifugation at 400× *g*. The precipitate was rinsed with deionized water.

### 2.3. Synthesis of Chitosan-Iron Oxide Nanoparticles

Initially, CS (1 g) was dissolved in acetic acid (1%). The CS mixture was added into MNPs suspension and sonicated for 18 h. The reaction mixture was centrifuged and rinsed three times with deionized water before dried it at 70 °C [20].

### 2.4. Synthesis of Phytic Acid-Chitosan-Iron Oxide Nanocomposite

Briefly, 2 g of phytic sodium salt was added into 100 mL of deionized water. The phytic sodium salt solution was then mixed with CS-MNPs. The mixture was vortexed vigorously for 24 h followed by centrifugation at 400× *g*. Subsequently, the precipitate was rinsed using deionized water before dried it at 60 °C [11].

### 2.5. Cell Lines

All cell lines were procured from American Type Culture Collection (Rockville, MD, USA). All grown medium were supplemented with FBS (10% (*v*/*v*)), 100 μg/mL streptomycin, and 100 IU/mL penicillin. All cell lines were placed at 5% CO_2_ atmosphere and 37 °C humidified atmosphere incubator.

### 2.6. Determination of Cytotoxicity Using MTT Assay

Human colorectal adenocarcinoma (HT-29), human colon carcinoma (HCT-116), human cervical cancer (HeLa), human gastric adenocarcinoma (HGT-1), human hepatocellular carcinoma (HepG2), and mouse fibroblast (BALB/c 3T3) cells were seeded in 96-well plates (1 × 10^5^ cells/well) and incubated overnight. Phy-CS-MNP nanocomposite (1.56–00 μg/mL) was added into the 96 wells of HT-29, HCT-116, HeLa, HGT-1, HepG2, and BALB/c 3T3 cells. Untreated BALB/c 3T3 and cancer cell lines were included. Following 24 h, 48 h, and 72 h of incubation, 20 µL (5 mg/mL) of 3-(4,5-dimethylthiazol-2-yl)-2,5-diphenyltetrazolium bromide (MTT) was added into each well followed by 2–4 h incubation. Lastly, an aliquot of 100 µL of dimethyl sulfoxide was added into each well. The absorbance was read at 570 nm using ELISA microplate reader (Tecan, Männedorf, Switzerland). The wavelength of 630 nm was used as a reference.

### 2.7. Determination of Cell Cycle Arrest

Cell cycle arrest was determined using CycleTEST PLUS DNA Reagent Kit (BD Biosciences Pharmingen, Franklin Lakes, NJ, USA), following the recommended instruction. The HT-29 cell line was seeded in 25 cm^2^ tissue culture flask (1 × 10^6^ cells). After an overnight incubation, HT-29 cells were exposed to 22.5, 45, and 90 μg/mL of Phy-CS-MNP nanocomposite for 72 h. Positive control (1.4 μg/mL of 5-fluorouracil (5-FU)) was included. Following incubation for 72 h, the HT-29 cell was centrifuged at 30× *g* for 5 min at room temperature followed by the addition of buffer solution. The HT-29 cells were mixed with 250 μL of trypsin buffer, 200 μL of RNase buffer and trypsin inhibitor, followed by incubation (10 min) at room temperature, respectively. Lastly, 200 μL of propidium iodide (PI) stain solution was added and incubated for 10 min at 4 °C. Data analysis and acquisition were evaluated using NovoCyte Flow Cytometer (ACEA Biosciences, Inc., San Diego, CA, USA) with NovoExpress^®^ software (1.3.0, ACEA Biosciences, Inc., San Diego, CA, USA, 2018).

### 2.8. Determination of Apoptosis by Annexin V-Propidium Iodide Staining

Early and late apoptotic cells were analyzed using Annexin V-FITC Apoptosis Detection Kit I (BD Biosciences Pharmingen, Franklin Lakes, NJ, USA), following the recommended instruction. The cells were seeded in 25 cm^2^ tissue culture flask (1 × 10^6^ cells). After an overnight incubation, the cells were exposed to 22.5, 45, and 90 μg/mL of Phy-CS-MNP nanocomposite for 72 h. Positive control (1.4 μg/mL of 5-FU) was included. The cells were trypsinized and rinsed twice with phosphate-buffered saline-1% bovine serum albumin-ethylenediaminetetraacetic acid and resuspended with 100 μL of 1× binding buffer. An aliquot of 10 μL of PI and 5 μL of fluorescein isothiocyanate (FITC) were added and allowed to react for 10 min. Lastly, the fluorescence of cells was measured after adding 400 μL of 1× binding buffer before evaluated using NovoCyte Flow Cytometer (ACEA Biosciences, Inc., San Diego, CA, USA) with NovoExpress^®^ software (1.3.0, ACEA Biosciences, Inc., San Diego, CA, USA, 2018).

### 2.9. Determination of Bax and Bcl-2 Activities 

Bax and Bcl-2 activities were quantified using Bax and Bcl-2 human SimpleStep ELISA^®^ Kits (Abcam, Cambridge, UK), following the recommended instruction. Initially, the cells were seeded in 25 cm^2^ tissue culture flask (1 × 10^5^ cells) and incubated overnight. The cells were exposed to 22.5, 45, and 90 μg/mL of Phy-CS-MNP nanocomposite and 1.4 μg/mL of 5-FU for 72 h. Following incubation for 72 h, the cells were centrifuged at 500× *g* for 5 min at 4 °C to discard the medium. The cells were washed two times with phosphate-buffered saline (PBS) and cold 1× Cell Extraction Buffer PTR, and incubated for 20 min on ice. The cell lysates were subsequently centrifuged at 18,000× *g* and 4 °C for 20 min. An aliquot of the sample was diluted to the desired concentration in 1× Cell Extraction Buffer PTR. Fifty µL of standard or sample was mixed with 50 µL of antibody cocktail in each well of a 96-well plate. The plate was sealed prior to incubation for 1 h at room temperature. Each well was rinsed with 3 × 350 µL 1× wash buffer PT. An aliquot of 100 µL of TMB Substrate was added into each well followed by 10 min incubation at 400× *g*. Subsequently, 100 µL of Stop Solution was added and incubated for 1 min before read it at the wavelength of 450 nm. Human Bax or Bcl-2 protein was used as a standard. The Bax standard stock solution (400 ng/mL) was prepared by adding 200 µL of deionized water and incubated for 10 min at room temperature. An aliquot of 225 µL of 1× Cell Extraction Buffer PTR was added into the tube number 1 and 150 µL of 1× Cell Extraction Buffer PTR was added into the tube numbers 2–8. Stock solutions were prepared using the dilution series. Standard tube number 8 contains no protein (blank control). The human Bcl-2 standard stock solution (200 ng/mL) was prepared by adding 1 mL of 1× Cell Extraction PTR and incubated at room temperature for 3 min. Standards 2–8 were added with 150 µL of 1× Cell Extraction Buffer PTR into each tube. A working dilution of Bcl-2 standard was prepared using dilution series. Standard tube number 8 contains no protein (blank control).

### 2.10. Caspase-3 and Caspase-8 Activities

The caspase-3 and -8 assays were evaluated using a colorimetric assay kit (R&D Systems, Minneapolis, MN, USA), following the recommended instruction. Initially, the cells were seeded in 6-well plates (1 × 10^5^ cells/well) and incubated overnight. The cells were then treated with 22.5, 45, and 90 μg/mL of Phy-CS-MNP nanocomposite and 1.4 μg/mL of 5-FU. Following 72 h of treatment, the cells were centrifuged at 250× *g* for 10 min to discard the medium. An aliquot of 25 μL of cold lysis buffer was added into the cell pellets. About 50 μL of 2× Reaction Buffer 8 or 2× Reaction Buffer 3 was mixed with 50 μL of cell lysate containing 200 μg of total protein, followed by 5 μL of caspase-8 or caspase-3 colorimetric substrate (IETD-*p*Na or DEVD-*p*Na). Both caspase-3 and -8 activities were assessed at a wavelength of 405 nm after 2 h incubation at 37 °C.

### 2.11. Total RNA Extraction and cDNA Synthesis

Ribonucleic acid was isolated using TRI Reagent^®^ (Sigma-Aldrich, St. Louis, MO, USA), following the recommended instruction. The cells were seeded in 25 cm^2^ culture flask (1 × 10^5^ cells) and incubated overnight. After treatment with different concentrations (22.5, 45, and 90 μg/mL) of Phy-CS-MNP nanocomposite and 1.4 μg/mL of 5-FU for 72 h, the cells were homogenized and the lysates were aliquot in microcentrifuge tubes. An aliquot of 1 mL TRI Reagent^®^ was added into 25 cm^2^ tissue culture flask and resuspended. The homogenized sample stands at room temperature for 5 min to allow dissociation of nuclear protein complexes. Hundred μL of 1-bromo-3-chloropropane per mL of TRI Reagent^®^ used was added and vortexed vigorously for 15 s followed by 2–15 min incubation at room temperature. The upper layer was precipitated after an addition of 500 μL of isopropanol. Following 5–10 min at room temperature, the sample was centrifuged at 11,500× *g* and 2–8 °C for 10 min. The RNA pellet was washed using 75% (*v*/*v*) ethanol before being centrifuged at 2–8 °C for 5 min. Subsequently, the RNA pellet was mixed with 50 μL of RNase free water and resuspended before stored at −80 °C. High Capacity RNA-to-cDNA Kit (Applied Biosystems, Foster City, CA, USA) was used for reverse-transcription, following the recommended instruction.

### 2.12. Quantitative Real-Time Polymerase Chain Reaction 

Table 1 shows the nucleotide primer sequences originating from human origin. Bio-Rad-iQ™ 5 Multicolor Real-Time PCR Detection System was used to determine the real-time PCR reaction. The analysis was performed using SYBR^®^ Select Master Mix (CFX), following the recommended instruction. All controls and samples were analyzed using Bio-Rad-iQ™ 5 Multicolor Real-Time PCR Detection System. The data analysis was determined using CFX Manager™ software, version 1.6 (Bio-Rad, Hercules, CA, USA, 2012). Housekeeping genes (glyceraldehyde-3-phosphate dehydrogenase (*GAPDH*), 18S rRNA, and beta-actin (*ACTB*)) were used for normalization.

### 2.13. Protein Extraction 

The protein was extracted with 3 μL of protease inhibitor cocktail and 300 μL of radioimmune precipitation assay (RIPA) lysis buffer. The cells were incubated at 4 °C for 1 h with agitation. The resultant lysates were centrifuged at 9300× *g* for 10 min and the supernatant containing proteins were collected and subsequently stored at −80 °C.

### 2.14. Western Blotting Analysis

Briefly, the protein (50 μg) was separated using electrophoresis at 10% sodium dodecyl sulfate-polyacrylamide gel electrophoresis (SDS-PAGE) with separating gel and stacking gel. The protein ladder and protein lysate were loaded onto the gels, and subsequently the gels were run with 1× running buffer (0.192 M glycine, 0.1% sodium dodecyl sulfate (SDS), and 0.025 M Tris base; pH 8.3) at 75 V for 15 min followed by 120 V for 45 min. The gel was soaked in 1× Towbin transfer buffer (20% (*v*/*v*) methanol, 190 mM glycine, and 25 mM Tris-base; pH 8.3) for 10 min at room temperature. The Mini Trans-Blot Filter Papers were rinsed in Towbin transfer buffer for 30 s, while polyvinylidene difluoride (PVDF) membrane was immersed in methanol for 30 s, followed by rinsing with Towbin transfer buffer. The protein was transferred to a PVDF membrane by running the transfer apparatus at 400 mA and 100 V for 2 h. Five percent of skim milk was used to block the PVDF membrane to saturate an unoccupied region of the membrane. Rabbit polyclonal to nuclear factor-kappa B (NF-ĸB) p65 and rabbit monoclonal [EPR1327] to cytochrome c (Abcam, Cambridge, UK) were used at a 1:10,000 dilution and incubated for 24 h at 4 °C. Lastly, the membranes were incubated with goat polyclonal secondary antibody to rabbit IgG conjugated to horseradish peroxidase (HRP) (Abcam, Cambridge, UK) at a 1:10,000 dilution for 1 h at room temperature. The protein bands were detected using a Clarity western ECL substrate and a chemiluminescence imager (Bio-Rad, Hercules, CA, USA). To confirm the protein concentrations in all loaded samples were equal, beta-actin (Abcam, Cambridge, UK) was utilized as a loading control. Image J Software (1.4.3.67, National Institute of Health, NIH, Bethesda, MD, USA, 2006) was used to evaluate the densitometric of the band intensity.

### 2.15. Statistical Analysis

The results were shown as mean ± SD. The statistical significance was assessed by one-way analysis of variance (ANOVA) using SPSS version 17.0 (IBM Corporation, Chicago, IL, USA, 2009). The data were considered significant when *p*-value less than 0.05.

## 3. Results 

### 3.1. Treatment with Phy-CS-MNP Nanocomposite Decreases the Viability of HT-29 Cells

**Figure 1:**
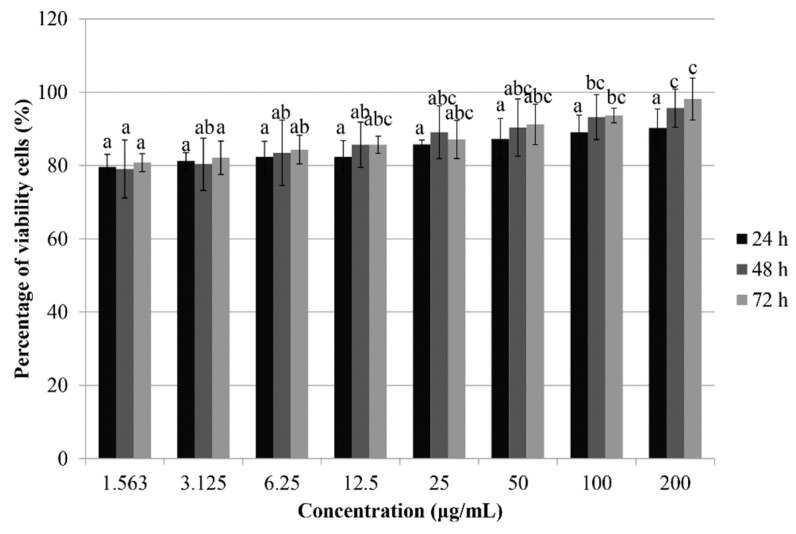
Treatment of Phy-CS-MNP nanocomposite in mouse fibroblast (BALB/c 3T3) cell lines evaluated using 3-(4,5-dimethylthiazol-2-yl)-2,5-diphenyltetrazolium bromide (MTT) assay. Values are reported as mean ± SD (n = 3). Value with different superscript letter indicates significant difference between groups by Tukey’s test (*p* < 0.05). Treatment with 200 μg/mL of Phy-CS-MNP nanocomposite for 48 h and 72 h significantly increased the survival of BALB/c 3T3 cell line compared to those treated with lower concentrations (1.563–6.25 μg/mL) (*p* < 0.05).

As shown in Table 2, treatment with Phy-CS-MNP nanocomposite decreases the viability of HCT-116 cells in a time-dependent manner after 24 h (157.13 ± 9.03 μg/mL), 48 h (120.24 ± 6.75 μg/mL), and 72 h (101.34 ± 12.23 μg/mL). Consistent with the cytotoxic effect observed in HCT-116 cells, Phy-CS-MNP nanocomposite also decreases the viability of HeLa cells in a time-dependent manner, with the IC_50_ values 134.96 ± 10.22, 130.77 ± 9.05, and 127.01 ± 5.11 μg/mL, after 24 h, 48 h, and 72 h, respectively. We found that, after treatment with Phy-CS-MNP nanocomposite for 72 h, both HGT-1 (76.92 ± 15.09 μg/mL) and HepG2 (80.11 ± 2.36 μg/mL) cells were inhibited. Further, treatment with Phy-CS-MNP nanocomposite also inhibited the viability of HT-29 cells after 24 h, 48 h, and 72 h, with IC_50_ value 112.71 ± 18.72, 79.33 ± 9.02, and 45.63 ± 5.77 μg/mL [11], respectively. The percentage of viability of the BALB/c 3T3 cell lines after treatment with Phy-CS-MNP nanocomposite was also evaluated using MTT assay. Our findings showed that the survival of the BALB/c 3T3 cell line was consistently greater than 75% (Figure 1).

### 3.2. Treatment with Phy-CS-MNP Nanocomposite Triggers G_0_/G_1_ Cell Cycle Arrest in HT-29 Cells

**Figure 2:**
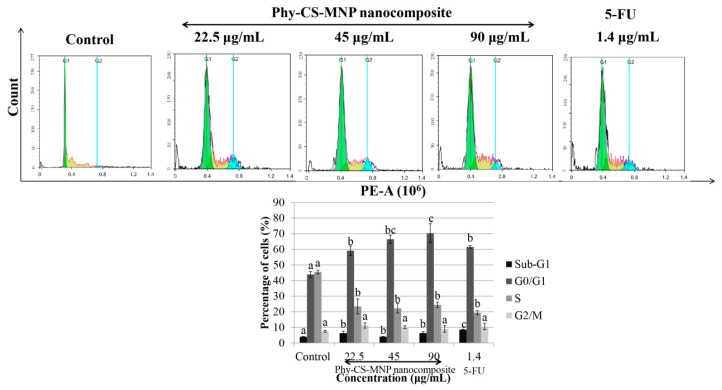
Cell cycle distribution of HT-29 cells treated with different concentrations of Phy-CS-MNP nanocomposite (22.5, 45, and 90 μg/mL) and 1.4 μg/mL of 5-FU for 72 h and analyzed using flow cytometry. Values are reported as mean ± SD (n = 3). Value with different superscript letter indicates significant difference between groups by Tukey’s test (*p* < 0.05). The percentage of cells in the sub-G_1_ phase was significantly increased at 22.5, 45, and 90 μg/mL of Phy-CS-MNP nanocomposite compared to the control (*p* < 0.05). Treatment with 22.5, 45, and 90 μg/mL of Phy-CS-MNP nanocomposite significantly increased the cell populations at G_0_/G_1_ phase compared to the control (*p* < 0.05) with a concomitant decreased of the S phase at 72 h.

Among all the groups presented in Figure 2, our cell cycle analysis showed that treatment with 1.4 μg/mL of 5-FU for 72 h significantly accumulated the percentage of cells at sub-G_1_ compared to untreated HT-29 cells (*p* < 0.05). Consistent with the effect observed in 5-FU, the sub-G_1_ phase was also significantly increased (*p* < 0.05) at 22.5, 45, and 90 μg/mL of Phy-CS-MNP nanocomposite compared to the control. Treatment with 22.5, 45, and 90 μg/mL of Phy-CS-MNP nanocomposite significantly increased (*p* < 0.05) the cell populations at G_0_/G_1_ phase compared to the control with a concomitant decreased of the S phase at 72 h (Figure 2).

### 3.3. Treatment with Phy-CS-MNP Nanocomposite Triggers Apoptosis in HT-29 Cells

**Figure 3:**
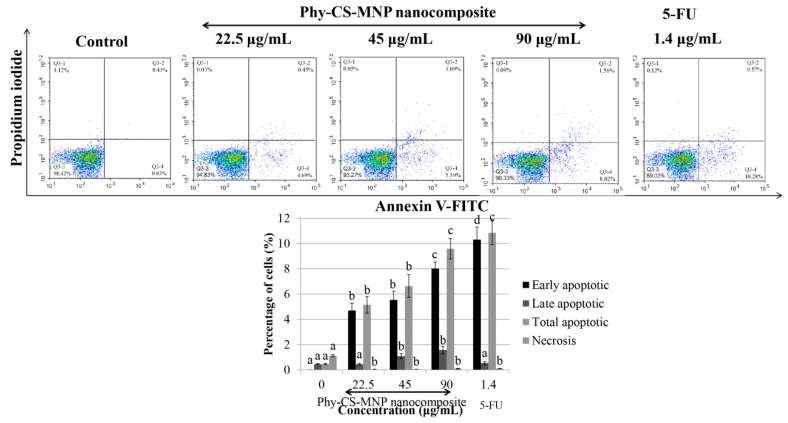
Apoptotic cell death of HT-29 cells treated with different concentrations of Phy-CS-MNP nanocomposite (22.5, 45, and 90 μg/mL) and 1.4 μg/mL of 5-FU for 72 h and analyzed using flow cytometry. Values are reported as mean ± SD (n = 3). Value with different superscript letter indicates significant difference between groups by Tukey’s test (*p* < 0.05). Treatment with 22.5, 45, and 90 μg/mL of Phy-CS-MNP nanocomposite significantly triggered the percentage of early apoptotic HT-29 cells compared to the control (*p* < 0.05). The percentage of late apoptotic cells was significantly increased after treated with 45 and 90 μg/mL of Phy-CS-MNP nanocomposite compared to the control (*p* < 0.05). Incubation with 22.5, 45, and 90 μg/mL of Phy-CS-MNP nanocomposite significantly increased the total apoptotic cells compared to the control (*p* < 0.05).

As depicted in Figure 3, treatment with 22.5, 45, and 90 μg/mL of Phy-CS-MNP nanocomposite significantly triggered the percentage of early apoptotic HT-29 cells compared to the untreated HT-29 cells (*p* < 0.05). Treatment with 45 and 90 μg/mL of Phy-CS-MNP nanocomposite significantly increased (*p* < 0.05) the percentage of late apoptotic cells compared to the control. Further, incubation with 22.5, 45, and 90 μg/mL of Phy-CS-MNP nanocomposite significantly increased (*p* < 0.05) the total apoptotic cells compared to the untreated HT-29 cells, with the highest effect noted at 90 μg/mL of Phy-CS-MNP nanocomposite (Figure 3).

### 3.4. Treatment with Phy-CS-MNP Nanocomposite Triggers Bax and Downregulates Bcl-2 Protein Expression in HT-29 Cells

**Figure 4:**
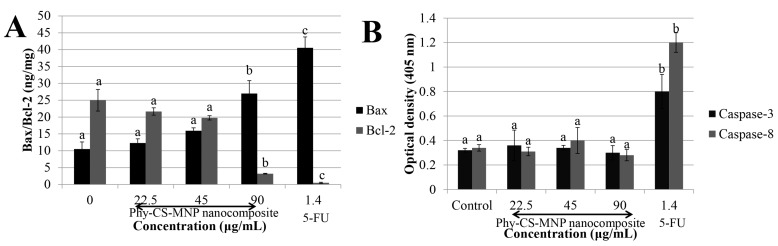
Apoptotic activities of different concentrations of Phy-CS-MNP nanocomposite (22.5, 45, and 90 μg/mL) and 1.4 μg/mL of 5-FU in HT-29 cells. Apoptotic protein expressions of (**A**) Bax and Bcl-2 and (**B**) Caspase-3 and -8 activities in HT-29 cells treated with different concentrations of Phy-CS-MNP nanocomposite (22.5, 45, and 90 μg/mL) and 1.4 μg/mL of 5-FU. Values are reported as mean ± SD (n = 3). Value with different superscript letter indicates significant difference between groups by Tukey’s test (*p* < 0.05). Treatment with 90 μg/mL of Phy-CS-MNP nanocomposite significantly upregulated Bax protein level compared to the control (*p* < 0.05). However, no significant difference in Bax protein expression between the untreated cells and those from the groups treated with 22.5 μg/mL or 45 μg/mL of Phy-CS-MNP nanocomposite (*p* > 0.05). Bcl-2 protein expression was significantly decreased at a concentration of 90 μg/mL compared to the control (*p* < 0.05). Treatment with 22.5 μg/mL and 45 μg/mL of Phy-CS-MNP nanocomposite did not significantly decrease Bcl-2 protein expression compared to the control (*p* > 0.05). Treatment with Phy-CS-MNP nanocomposite was not significantly increased the caspase-3 and -8 activities compared to the untreated cells (*p* > 0.05).

Our analysis revealed that treatment with 90 μg/mL of Phy-CS-MNP nanocomposite significantly upregulated Bax protein level compared to the untreated HT-29 cells (*p* < 0.05). Treatment with 22.5 μg/mL and 45 μg/mL of Phy-CS-MNP nanocomposite was not significantly decreased the Bcl-2 protein level compared to the control (*p* > 0.05). In contrast, the Bcl-2 protein expression was reduced after treatment with 90 μg/mL of Phy-CS-MNP nanocomposite (Figure 4A).

### 3.5. Treatment with Phy-CS-MNP Nanocomposite Does Not Activate Caspase-3 and -8 Activities in HT-29 Cells

As presented in Figure 4B, the cells treated with Phy-CS-MNP nanocomposite did not significantly increased caspase-3 and -8 activities compared to the untreated cells (*p* > 0.05). Expectedly, treatment with 1.4 μg/mL of 5-FU significantly activated caspase-3 and -8 activities compared to the untreated cells (*p* < 0.05) (Figure 4B).

### 3.6. Treatment with Phy-CS-MNP Nanocomposite Downregulates Cdk4 and Upregulates JNK1 and iNOS mRNA Expression in HT-29 Cells 

**Figure 5:**
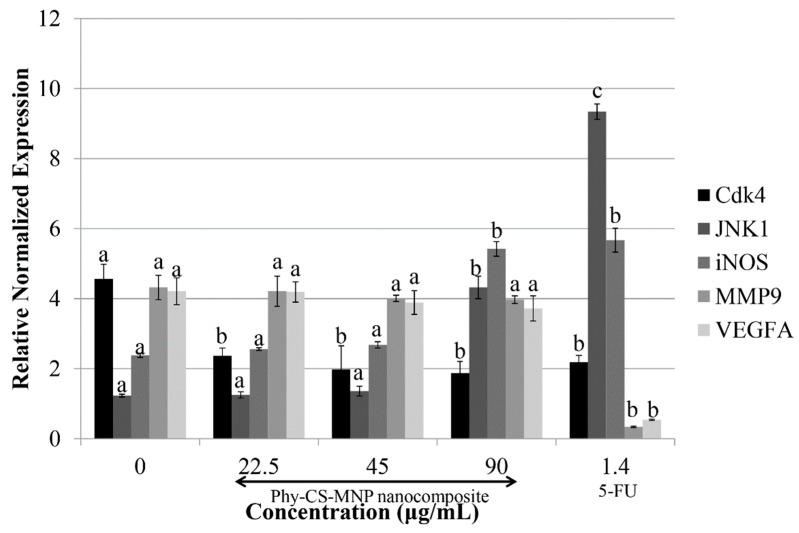
Expression of *Cdk4*, *JNK1*, *iNOS*, *MMP9*, and *VEGFA* at mRNA levels in HT-29 cells incubated with different concentrations of Phy-CS-MNP nanocomposite (22.5, 45, and 90 μg/mL) and 1.4 μg/mL of 5-FU for 72 h and analyzed using quantitative real-time PCR. Values are reported as mean ± SD (n = 3). Value with different superscript letter indicates a significant difference between groups by Tukey’s test (*p* < 0.05). The mRNA level of *Cdk4* was significantly reduced after treated with Phy-CS-MNP nanocomposite (*p* < 0.05). The mRNA expression of *JNK1* was significantly upregulated at 90 μg/mL of Phy-CS-MNP nanocomposite (*p* < 0.05). Similarly, *iNOS* expression was also significantly upregulated at 90 μg/mL of Phy-CS-MNP nanocomposite compared to the control (*p* < 0.05). However, no significant difference between the control and those from the groups treated with 22.5 μg/mL or 45 μg/mL of Phy-CS-MNP nanocomposite (*p* > 0.05). The transcriptional activity of *VEGFA* and *MMP9* in HT-29 cells treated with Phy-CS-MNP nanocomposite was not significantly different compared to the control (*p* > 0.05). (Abbreviations: *Cdk4*, cyclin-dependent kinase 4; *iNOS*, inducible nitric oxide synthase; *JNK1*, c-jun N terminal kinase 1; *MMP9*, matrix metallopeptidase 9; *VEGFA*, vascular endothelial growth factor A.)

Our data showed that the transcriptional activity of *Cdk4* was reduced accordingly when the concentration of Phy-CS-MNP nanocomposite was increased. The mRNA expression of *JNK1* was significantly upregulated (*p* < 0.05) at 90 μg/mL of Phy-CS-MNP nanocomposite compared to the control. Similarly, *iNOS* expression was also significantly upregulated (*p* < 0.05) at 90 μg/mL of Phy-CS-MNP nanocomposite compared to the control (Figure 5). Yet, transcriptional activity of *VEGFA* and *MMP9* in HT-29 cells treated with Phy-CS-MNP nanocomposite was not significantly different (*p* > 0.05) compared to the control (Figure 5). In comparison to the untreated HT-29 cells, 5-FU (positive control) suppressed the *MMP9* and *VEGFA* mRNA expression, significantly (*p* < 0.05) (Figure 5).

### 3.7. Treatment with Phy-CS-MNP Nanocomposite Upregulates the Protein Levels of Cytochrome c and NF-κB 

**Figure 6:**
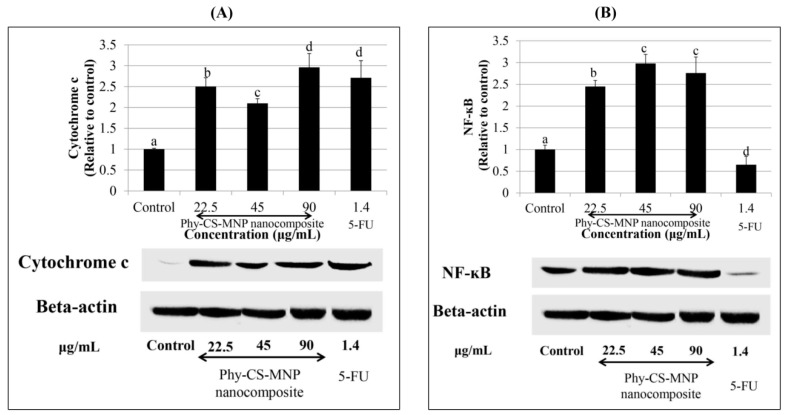
Protein expressions of (**A**) cytochrome c and (**B**) *NF-κB* in HT-29 cells incubated with different concentrations of Phy-CS-MNP nanocomposite (22.5, 45, and 90 μg/mL) and 1.4 μg/mL of 5-FU for 72 h and analyzed using western blotting. Values are reported as mean ± SD (n = 3). Values with different superscript letters indicate significant differences between groups by Tukey’s test (*p* < 0.05). The control (beta-actin) images are re-used for illustrative purposes. Protein expression of cytochrome c was significantly increased in the cytosol, with the maximum effect observed at the concentration of 90 μg/mL of Phy-CS-MNP nanocomposite. However, very low cytochrome c protein expression was observed in the untreated HT-29 cells (control). We found that the Phy-CS-MNP nanocomposite was significantly upregulated the *NF-κB* protein expression compared to the control (*p* < 0.05). (Abbreviation: *NF-κB*, nuclear factor-kappa B).

The result showed that the cytochrome c protein level was increased significantly in the cytosol, with the maximum effect observed at the concentration of 90 μg/mL of Phy-CS-MNP nanocomposite (Figure 6A). However, very low cytochrome c protein expression was noted in the control. The *NF-κB* protein expression was upregulated after treated with Phy-CS-MNP nanocomposite (Figure 6B).

## 4. Discussion 

Phytic acid has been shown to be effective in the prevention of diseases [21,22]. Although phytic acid has tremendous potential as a therapeutic agent for cancer, the therapeutic application of phytic acid might be impeded by its shortcomings including low bioavailability and poor stability which has caused administration at unrealistic therapeutic dosage. Nanotechnology has been utilized for protection, controlled release of bioactive compounds, and encapsulation. Nanotechnology could be used as a carrier for cancer therapy because most of the processes occur at the nanoscale level [23].

The characterization of Phy-CS-MNP nanocomposite has been performed in our earlier study to evaluate the characteristic of the nanoparticle. Powder X-ray diffraction (PXRD) analyses revealed that the process of coating did not alter the MNPs phase changes; the position of the peaks remains the same [24]. By contrast, the coating process caused crystallinity and a lower peak intensity of the Phy-CS-MNP nanocomposite [11]. The mean particle size of MNPs was ~8 nm, calculated from the Debye–Scherrer equation [11]. Based on the FTIR spectra analyses, MNPs demonstrated an absorption peak at 569 cm^−1^ because of the stretching of Fe–O in Fe_3_O_4_. This peak was shifted to 565 cm^−1^ in the Phy-CS-MNP nanocomposite, indicating the presence of MNPs in the nanocomposites [25]. The characteristic band for CS was observed at 1560 cm^−1^, which confirms that the MNPs were successfully coated with CS [26]. These data demonstrated that MNPs are bound with CS via glycosidic bonds, whereas phytic acid bound with CS polymer via hydrogen bonds [9,27]. The UV-Vis absorption spectroscopy further demonstrated that 12.9% of phytic acid was loaded into Phy-CS-MNP nanocomposite [11].

There are several kinetic models demonstrating the total release of phytic acid from Phy-CS-MNP nanocomposite. Three general kinetic models have been used including parabolic diffusion, pseudo-first-order, and pseudo-second-order models. The parabolic diffusion model is shown as follows [28]:

(1 − *M_t_*/*M_o_*) = *kt*^−0.5^ + *bt*

*M_t_* = Phytic acid content remains in the nanocomposite at release time *t*; *M_o_* = Phytic acid content remains in the nanocomposite at release time 0; Pseudo-first-order kinetic equation is described as follows [29]:

In (*q_e_* − *q_t_*) = In *q_e_* − *kt*

*q_t_* = Release amount at time *t*; *q_e_* = Equilibrium release amount; *k* = The constant of the corresponding release rate

The release behavior of drugs from nanocomposite in the pseudo-second-order kinetic model is written as followed [30]:

*t*/*q_t_* = 1/*kq*^2^*_e_* + *t*/*q_e_*

A straight line was obtained by plotting *t*/*q_t_* against *t*. The release rate constant *k* and *q_e_*, can be calculated using the following equation:

*k* = 1/*q*^2^*_e_* · intercept

Among all kinetic models described above, we discovered that the pseudo-second-order model well-governed the release kinetic processes of phytic acid from Phy-CS-MNP nanocomposite at pH 7.4 and pH 4.8, with release rate constant (*k*) values of 1.69 × 10^−5^ and 2.86 × 10^−5^ mg/min and correlation coefficients of (*R*^2^) 0.9996 and 0.9980, respectively [11]. About 86% and 93% of phytic acid could be released within 127 h and 56 h using pH 7.4 and pH 4.8 phosphate buffer solution, respectively governed by pseudo-second-order kinetic model [11].

To evaluate the antiproliferative effect of Phy-CS-MNP nanocomposite on cancer cells, HCT-116, HeLa, HT-29, HGT-1, HepG2, and BALB/c 3T3 cells were exposed to different concentrations of Phy-CS-MNP nanocomposite (1.56–200 μg/mL) for 24 h, 48 h, and 72 h, and the cytotoxic effect was measured using the MTT assay. We observed that the HT-29 cells were relatively more sensitive after treatment with Phy-CS-MNP nanocomposite compared to other cancer cell lines studied. It suppressed the viability of HT-29 cells in a time-dependent manner, with IC_50_ value 112.71 ± 18.72, 79.33 ± 9.02, and 45.63 ± 5.77 μg/mL [11] for 24 h, 48 h, and 72 h, respectively. Importantly, no cytotoxicity was found in Phy-CS-MNP nanocomposite-treated BALB/c 3T3 cells as evaluated by MTT assay. Collectively, our data suggest that Phy-CS-MNP nanocomposite can induce cytotoxicity in different cancer cells, in which HT-29 cells are the most sensitive compared to other cancer cell lines studied. Therefore, the HT-29 cell line was selected for further analyses. Based on the IC_50_ value of HT-29 cells after 72 h treatment with Phy-CS-MNP nanocomposite, these three concentrations (22.5, 45, and 90 μg/mL) were selected for further analyses. As a positive control, the HT-29 cell line was incubated with the commercial drug, 5-FU. The IC_50_ values of 5-FU against HT-29 cells at 72 h were 1.40 ± 0.65 μg/mL as evaluated using the MTT assay [31].

To verify whether Phy-CS-MNP nanocomposite-induced growth suppression in HT-29 cells is modulated by cell cycle arrest, the HT-29 cell line was incubated with Phy-CS-MNP nanocomposite for 72 h and measured using flow cytometry. Our results demonstrated that sub-G_1_ phase was increased significantly at 22.5, 45, and 90 μg/mL of Phy-CS-MNP nanocomposite, which indicates that Phy-CS-MNP nanocomposite induces a population of sub-G_1_ phase following treatment with Phy-CS-MNP nanocomposite, suggest that DNA degradation due to the activation of endogenous nucleases during apoptosis [32]. Treatment with 22.5, 45, and 90 μg/mL of Phy-CS-MNP nanocomposite significantly increased (*p* < 0.05) the cell populations in G_0_/G_1_ phase compared to the control with a concomitant decreased of the S phase at 72 h. This result implies that Phy-CS-MNP nanocomposite regulates several biological processes associated with cell survival and death. Our findings presented in this study demonstrated that Phy-CS-MNP nanocomposite destroys HT-29 cells in dividing state.

To evaluate if the cytotoxic activity of Phy-CS-MNP nanocomposite was due to the induction of apoptosis, HT-29 cells were exposed to 22.5, 45, and 90 μg/mL of Phy-CS-MNP nanocomposite for 72 h and evaluated by Annexin V-FITC/PI double staining using flow cytometry. Our data revealed that incubation with Phy-CS-MNP nanocomposite for 72 h triggered the apoptotic HT-29 cells. This finding implied that induction of apoptotic cell death in HT-29 cells by Phy-CS-MNP nanocomposite could be of greater significance in colon cancer. Accordingly, Phy-CS-MNP nanocomposite might be used as a potential therapeutic agent for human colorectal cancer.

Mitochondria play a critical role in apoptosis by decreasing transmembrane potential, generation of ROS, increasing the outer mitochondrial membrane permeability, and releasing of cytochrome c [33,34]. Anti-apoptotic Bcl-2 family members can inhibit these phenomenon, while pro-apoptotic Bcl-2 family members such as Bad, Bak, and Bax may promote mitochondrial events. The study showed that treatment with 90 μg/mL of Phy-CS-MNP nanocomposite resulted in the elevation of mitochondria Bax expression. However, Bcl-2 expression was downregulated in the concentration of 90 μg/mL of Phy-CS-MNP nanocomposite. The reason for the lack of any clear concentration-dependent effects needs to be further explored. One of the possible reasons may be due to the efficiency of Phy-CS-MNP nanocomposite involved in the upregulation of Bax and downregulation of Bcl-2 expression is reached in the concentration of 90 μg/mL, implied that higher concentration of Phy-CS-MNP nanocomposite may confer better functional properties in the modulation of Bax and Bcl-2 expression. However, we found that no significant difference in Bax and Bcl-2 expression between control and those of the cells treated with 22.5 or 45 μg/mL of Phy-CS-MNP nanocomposite (*p* > 0.05). These data may reveal that low concentrations (22.5 and 45 μg/mL) of Phy-CS-MNP nanocomposite are insufficient to stimulate the activation of Bax and Bcl-2 expression. In the current study, treatment with Phy-CS-MNP nanocomposite resulted in cytochrome c release from the mitochondria to the cytosol. Cytochrome c release from mitochondria that occurs after treatment with 90 μg/mL of Phy-CS-MNP nanocomposite is possibly controlled by Bax. Substantial evidence has demonstrated that the translocation of Bax to mitochondria can modify the permeability of the outer mitochondrial membrane, subsequently leading to the release of cytochrome c to the cytosol [35] and triggering a caspase cascade from apical caspases to effector caspases, which ultimately leads to apoptotic cell death. Taken together, sufficient Bax resides in the mitochondrial membrane may trigger cytochrome c release after treatment with 90 μg/mL of Phy-CS-MNP nanocomposite.

Caspases are cysteine proteases which play a major role in the execution phase of apoptosis [36]. Our data showed that Phy-CS-MNP nanocomposite did not activate caspase-3 and -8 activities, suggesting that Phy-CS-MNP nanocomposite triggers a caspase-independent signal transduction pathway. The observed effects are in parallel with the result obtained by Tor et al. [37], who revealed that ethyl acetate extract of *Dillenia suffruticosa* triggered apoptosis in MCF-7 cells via a caspase-independent pathway. Stimulation of caspases may be imperative but not exclusive in the induction of apoptosis [38]. Execution of apoptosis can be triggered without the presence of caspases. The induction of apoptosis can be facilitated by non-caspase proteases such as endonuclease, proteases, and cathepsin [39]. Moreover, exposure of chromatin condensation and phosphatidylserine are not mandatorily followed by the elevation of caspase effector [40].

To confirm that the cell cycle was arrested at the G_0_/G_1_ phase, we analyzed the transcriptional activity of *Cdk4* in the Phy-CS-MNP nanocomposite-treated HT-29 cells by quantitative real-time PCR. The data demonstrated that Phy-CS-MNP nanocomposite treated HT-29 cells reduced the *Cdk4* mRNA expression in a concentration-dependent pattern, indicating that the cells were blocked at the G_0_/G_1_ phase [41].

To further verify whether Phy-CS-MNP nanocomposite could suppress the viability of HT-29 cells, we determined the chemoprevention mechanism of *JNK1* mRNA level on Phy-CS-MNP nanocomposite in this model. Upregulation of JNK has been associated with the activation of apoptosis in several cancers [42,43]. This is in accordance with our observations, in which 90 μg/mL of Phy-CS-MNP nanocomposite activates the transcriptional activity of *JNK1*. This result suggests that *JNK1* expression is involved in the Phy-CS-MNP nanocomposite-driven apoptotic process and thereby inhibiting the proliferation of HT-29 cells. Therefore, our results implied that there is an important link between *JNK1* expression and apoptosis modulation.

Additionally, the roles of *iNOS* in the inhibition of colorectal cancer elicited by Phy-CS-MNP nanocomposite also require further elucidation. The involvement of *iNOS* is also crucial in the modulation of cancer cell proliferation. Hence, the transcriptional activity of *iNOS* in HT-29 cells was evaluated to assess whether Phy-CS-MNP nanocomposite could modulate *iNOS* at the mRNA level. Similar to *JNK1* expression, a marked elevation was also observed in the transcriptional activity of *iNOS* after treatment with 90 μg/mL of Phy-CS-MNP nanocomposite. The modulation of *JNK1* and *iNOS* mRNA expression is observed at 90 μg/mL of Phy-CS-MNP nanocomposite suggesting that a higher concentration of Phy-CS-MNP nanocomposite may confer better functional properties in the modulation of *JNK1* and *iNOS* transcriptional activity in HT-29 cells. These findings revealed that high levels of *iNOS* may inhibit the proliferation of colorectal cancer. In accordance with this, overexpression of *iNOS* was also found to be associated with the suppression of colon tumors [44]. These results suggest that Phy-CS-MNP nanocomposite reduced cancer proliferation via an anti-inflammatory mechanism involving *iNOS* expression. However, no significant difference was found in *iNOS* expression between the untreated cells and those of the cells treated with 22.5 or 45 μg/mL of Phy-CS-MNP nanocomposite (*p* > 0.05). Therefore, our results implied that Phy-CS-MNP nanocomposite did not block the reduction of cell proliferation at 22.5 and 45 μg/mL in this signaling pathway.

Concerning the potential metastatic signaling mediated by Phy-CS-MNP nanocomposite, real-time PCR analyses revealed that Phy-CS-MNP nanocomposite induces caspase-independent apoptosis and did not suppressed the transcriptional activity of *MMP9* and *VEGFA*, indicating that Phy-CS-MNP nanocomposite action was not likely mediated *VEGFA* or *MMP9* expression.

*NF-κB* was characterized as a crucial regulator in response to viruses and pathogens [45]. *NF-κB* modulates genes involved in numerous biological processes, for instance cell growth, inflammation, cell survival, and cell differentiation [46]. Despite compelling research evidence has revealed that *NF-κB* as a tumor-promoting transcription factor, several studies have unraveled *NF-κB* as one of the critical players in apoptosis [47,48]. These data suggest that *NF-κB* could be a pro- or anti-apoptotic protein. In the present study, treatment with Phy-CS-MNP nanocomposite in HT-29 cells upregulated *NF-κB* protein expression, which is in parallel with the results obtained by Kasibhatla et al. [49], who demonstrated that activation of *NF-κB* expression was associated with apoptosis and may trigger certain apoptosis-related genes. Numerous studies reported by Aoki et al. [50] also highlighted the pro-apoptotic effect possessed by *NF-κB* in response to oxidative stress. The anti-apoptotic or pro-apoptotic activity of *NF-κB* is highly dependent on the stimuli received and transcriptional activity modulation [51]. ROS triggers stimulation of *NF-κB* through the dissociation of IκB and subsequently resulting *NF-κB* to enter the nucleus and promote transcription by interacting with DNA [52]. Nonetheless, the upregulation of *NF-κB* in the present study remains to be elucidated.

## 5. Conclusions

This study clearly showed that Phy-CS-MNP nanocomposite induces cell cycle arrest at the G_0_/G_1_ phase and apoptosis in colorectal cancer through regulation of several signaling pathways, with the highest efficiency found at 90 μg/mL of Phy-CS-MNP nanocomposite. Our study demonstrated that Phy-CS-MNP nanocomposite modulates a caspase-independent pathway via the collapse of mitochondrial membranes. However, to fully elucidate the potential of Phy-CS-MNP nanocomposite as an anti-cancer agent, further studies are warranted to provide valuable insights for the treatment of human colorectal cancer and other human malignancies. Taken together, this finding provides substantial evidence that Phy-CS-MNP nanocomposite induces apoptosis and G_0_/G_1_ cell cycle arrest in the HT-29 cell line via modulation of intrinsic mitochondrial pathways.

## Figures and Tables

**Table 1:** The nucleotide sequence of PCR primers for amplification and sequence-specific detection of cDNA (obtained from the GenBank database).

Primer Name [Accession Number]	Oligonucleotides (5′-3′) Sequence
*Cdk4* [NM_000075.3]	F–GAAACTCTGAAGCCGACCAG R–AGGCAGAGATTCGCTTGTGT
*JNK1* [NM_139046.3]	F–GTGATCAATGGCTCTCAGCA R–TGACTAACCGACTCCCCATC
*iNOS* [AF049656.1]	F–GTGGTGACAAGCACATTTGG R–GTCATGAGCAAAGGCACAGA
*MMP9* [NM_004994.2]	F–GACAAGAAGTGGGGCTTCTG R–GCCATTCACGTCGTCCTTAT
*VEGFA* [NM_001287044.1]	F–CCCACTGAGGAGTCCAACAT R–AAATGCTTTCTCCGCTCTGA
*ACTB*^a^ [NM_001101.3]	F–AGAGCTACGAGCTGCCTGAC R–AGCACTGTGTTGGCGTACAG
*GAPDH*^a^ [NM_002046.4]	F–GGATTTGGTCGTATTGGGC R–TGGAAGATGGTGATGGGATT
18S rRNA ^a^ [HQ387008.1]	F–GTAACCCGTTGAACCCCATT R–CCATCCAATCGGTAGTAGCG

Abbreviations: *ACTB*, beta-actin; *Cdk4*, cyclin-dependent kinase 4; *GAPDH*, glyceraldehyde-3-phosphate dehydrogenase; *iNOS*, inducible nitric oxide synthase; *JNK1*, c-Jun N-terminal kinase 1; *MMP9*, matrix metallopeptidase 9; *VEGFA*, vascular endothelial growth factor A. ^a^ Housekeeping gene.

**Table 2:** Treatment of Phy-CS-MNP nanocomposite (1.56–200 μg/mL) on selected cancer cell lines for 24 h, 48 h, and 72 h evaluated by MTT assay.

Cancer Cell Lines	24 h	48 h	72 h
HT-29	112.71 ± 18.72 ^a^	79.33 ± 9.02 ^b^	45.63 ± 5.77 ^c^
HCT-116	157.13 ± 9.03 ^a^	120.24 ± 6.75 ^b^	101.34 ± 12.23 ^b^
HeLa	134.96 ± 10.22 ^a^	130.77 ± 9.05 ^a^	127.01 ± 5.11 ^a^
HGT-1	176.11 ± 13.19 ^a^	154.07 ± 12.33 ^a^	76.92 ± 15.09 ^b^
HepG2	98.05 ± 5.23 ^a^	82.13 ± 6.56 ^b^	80.11 ± 2.36 ^b^

Abbreviations: HCT-116, human colon carcinoma; HeLa, human cervical cancer; HepG2, human hepatocellular carcinoma; HGT-1, human gastric adenocarcinoma; HT-29, human colorectal adenocarcinoma; MTT, 3-(4,5-dimethylthiazol-2-yl)-2,5-diphenyltetrazolium bromide. Values are reported as mean ± SD (n = 3). Value with different superscript letter in the same row indicates significant difference between time points by Tukey’s test (*p* < 0.05). Treatment with Phy-CS-MNP nanocomposite for 72 h significantly reduced the viability of HT-29 cells compared to 24 h and 48 h (*p* < 0.05).
